# Examining Inequities in Clinical Outcomes for Indigenous Patients Treated With Dialysis in Canada: A Scoping Review

**DOI:** 10.1016/j.xkme.2025.101106

**Published:** 2025-09-16

**Authors:** Noémie Laurier, Taylor Stoesz, Andrea Quaiattini, Caitlin Gilpin, Romina Pace, Laura Horowitz, Rita S. Suri, Shaifali Sandal, Emilie Trinh

**Affiliations:** 1 McGill University, Faculty of Medicine and Health Sciences, Montreal, PQ, Canada; 2 McGill University, Institute of Health Sciences Education, Montreal, PQ, Canada; 3 Schulich Library of Physical Sciences, Life Sciences, and Engineering, McGill University, Montreal, PQ, Canada; 4 McGill University, Faculty of Education, Montreal, PQ, Canada; 5 Cree Board of Health and Social Services of James Bay, PQ, Canada; 6 Research Institute of the McGill University Health Center, Montreal, PQ, Canada; 7 Division of General Internal Medicine, McGill University, Montreal, PQ, Canada; 8 Division of Nephrology, McGill University, Montreal, PQ, Canada; 9 Division of Clinical and Translational Research, Department of Medicine, McGill University, Montreal, PQ, Canada

## Abstract

The burden of kidney disease among Indigenous peoples in Canada is disproportionately higher than the rest of the population. We aimed to synthesize the existing knowledge on the clinical outcomes among Indigenous peoples treated with dialysis in Canada. We searched MEDLINE, Embase, CINAHL, Scopus, Web of Science Core Collection, and the Bibliography of Indigenous Peoples of North America, supplemented by a gray literature review. The following inclusion criteria were used: (1) studies assessing dialysis patients, (2) including Canadian Indigenous patients, and (3) relating to incidence, mortality, treatment complications, access to care, and/or quality of life. Forty-four studies, conducted across multiple Canadian provinces, were included. Fifteen studies highlighted the higher prevalence of diabetic and nondiabetic kidney failure among Indigenous Canadians compared with non-Indigenous Canadians. Indigenous patients experienced more frequent dialysis-related infections and cardiovascular complications, increased hospitalization rates, lower rates of arteriovenous fistula creation, lower use of home dialysis, reduced access to health care services, and decreased quality of life because of relocation for dialysis. Few studies explored the underlying causes of the observed inequities. Our findings underscore the need to better understand the contributing factors to develop culturally appropriate interventions, codesigned with Indigenous communities, that promote equitable care for Indigenous patients receiving dialysis.

## Introduction

Indigenous peoples in Canada face significant health disparities, marked by poorer overall health outcomes, limited access to health care services and a higher burden of chronic diseases compared with non-Indigenous Canadians.^1^^,^^2^ These disparities are rooted in the enduring impacts of colonialism, including the multigenerational trauma from colonial practices, and the imposition of colonial institutions, including residential schools.^3^ As a result, colonialism has fueled persistent systemic inequities in health care, social, political, and economic systems, thus fostering deep mistrust toward Western medicine.^4^, ^5^, ^6^ Compounding these issues is the pervasive presence of anti-Indigenous racism in health care, which operates at structural, systemic, and individual levels.^7^

Recognizing and addressing these inequities has been identified as an urgent priority by stakeholders.^8^ Notably, the recent establishment of the Indigenous Health Equity Fund, allocating $2 billion CAD, reflects a growing commitment to support initiatives that promote health equity and improve outcomes for Indigenous peoples.^9^ To advance culturally safe care, it is essential to integrate Indigenous knowledge systems into health care policy and practice. Approaches such as Two-Eyed Seeing—which emphasizes the strengths of both Indigenous and Western ways of knowing—offer a powerful framework for reimagining health care delivery in Indigenous communities.^10^

In Canada, the number of patients requiring long-term dialysis has increased by 24% in the last 10 years, and the rate of dialysis start continues to increase every year.^11^ This growing demand imposes a significant economic burden on the Canadian health care system, as hemodialysis costs on average 60,000 CAD$ per patient per year in tertiary care centers and 45,000 CAD$ when delivered at home.^12^ Moreover, Indigenous peoples, who represent 5% of the Canadian population,^13^ are more likely to develop kidney failure compared with non-Indigenous people.^14^ Unfortunately, Indigenous patients of Canada with kidney failure are less likely to receive kidney transplantation than non-Indigenous Canadians.^14^

This scoping review aimed to characterize the existing knowledge on clinical outcomes among Indigenous peoples treated with dialysis in Canada, with a focus on identifying disparities between Indigenous and non-Indigenous peoples.

## Methods

This review was conducted using the scoping review approach proposed by Arksey and O’Malley^15^ further refined by Levac et al^16^ and Daudt et al^17^ and is reported using the Preferred Reporting Items for Systematic Review and Meta-Analysis for Scoping Reviews (Table S1).^18^ A Cree-origin research associate (C.G.) was involved to review the entire article and provide insight from an Indigenous stakeholder perspective.

### Eligibility Criteria

Articles were included if they (1) assessed patients with kidney failure (defined as estimated glomerular filtration rate < 15 mL/min/1.73 m^2^ or undergoing dialysis); (2) included Canadian Indigenous patients (First Nations, Inuit, and Metis); and (3) related to incidence, mortality, treatment complications, access to care and/or quality of life. Studies focusing exclusively on kidney transplant recipients were excluded, as this topic has already been addressed in a previous scoping review.^14^ As the purpose of this scoping review was to characterize existing evidence and identify knowledge gaps on the topic, the quality of the included studies was not formally assessed. Detailed inclusion and exclusion criteria are provided in Table S2.

### Search Strategy

A comprehensive search was conducted by a health sciences librarian (A.Q.) in MEDLINE(R) ALL (Ovid) using a combination of headings pertaining to the following topics: “End-stage renal disease,” “Indigenous,” “Canada,” and all related terms. No limits were used. The search strategy was then translated to 5 other databases: Embase Classic+Emabse (Ovid), CINAHL Plus with Fulltext (Ebsco), Scopus, Web of Science Core Collection, and Bibliography of Indigenous Peoples of North America (Ebsco) (see Items S1 and S2 for the complete search strategies). Searches were conducted from the databases' inception to July 15, 2024.

A supplementary Google Scholar search was also conducted using the keywords “[(end-stage kidney disease) OR (dialysis)] AND (Indigenous) AND (Canada) AND [(hospitalization) OR (mortality) OR (modality) OR (quality of life) OR (comorbidity)]”. Our supplementary search applied no restrictions on study type, encompassing journal articles, ongoing research projects, non-peer-reviewed literature and articles, conference abstracts, theses, and dissertations. The first 20 pages on Google Scholar were searched between July 1 and July 24, 2024. Finally, a gray literature search was conducted using 3 sources: Statistics Canada, the Government of Canada’s website and the Canadian Institute for Health Information, and involved a review of health boards, government reports, statistical reports, policy statements, reports from nongovernmental organizations and professional association websites. All 119 results under “Health and well-being” of Indigenous peoples on Statistics Canada were screened,^19^ as well as the 253 publications retrieved by using the search strategy “(Indigenous) AND (health)” on the Government of Canada website.^20^ The 2 reports, available at the time of the study conduct, on the Canadian Institute for Health Information under “First Nations, Inuit and Métis health” were also screened.^21^^,^^22^ To ensure the relevance and credibility of gray literature, we included only sources published by reputable organizations that reported outcomes aligned with the objectives of this scoping review.

### Screening of Records

The initial database search yielded 1,804 results, with 495 duplicates removed, resulting in 1,309 titles and abstracts to be screened. The gray literature search yielded no results, but supplementary searching identified one additional result.^23^ Screenings and full-text reviews were independently conducted by N.L. and T.S. Disagreements between reviewers were resolved by a third reviewer (E.T.) who independently reviewed the cases and made the final determination on study inclusion or exclusion.

### Outcomes

Outcomes of interest included kidney failure incidence rates, mortality rates, home dialysis use and complications, vascular access use and complications, access to care, hospitalization, and quality of life. These were carefully selected because they reflect key stages and challenges across the dialysis care trajectory and have been previously identified in the literature as important measures.^24^ Importantly, these outcomes were defined a priori and were considered equally in evaluating disparities and informing future interventions.

### Data Extraction and Analysis

Data extraction was performed independently by 2 reviewers (T.S and N.L) using a standardized extraction form developed and piloted before full extraction. The form captured key study characteristics, population details, outcomes of interest, and relevant findings. To ensure accuracy and consistency, both reviewers independently extracted data, and after every 10 articles, the extracted data were compared and discrepancies were discussed and resolved through consensus. If disagreements arose during the full extraction phase, they were resolved through discussion or by consulting a third reviewer (E.T.) when consensus could not be reached. This process ensured data quality and reliability throughout the review. Following data extraction, the data were systematically categorized according to the 7 predefined outcomes of interest. The results were analyzed and interpreted narratively; thematic analysis was not applicable because of the focus on summarizing quantitative and descriptive data rather than qualitative data.

## Results

**Figure 1:**
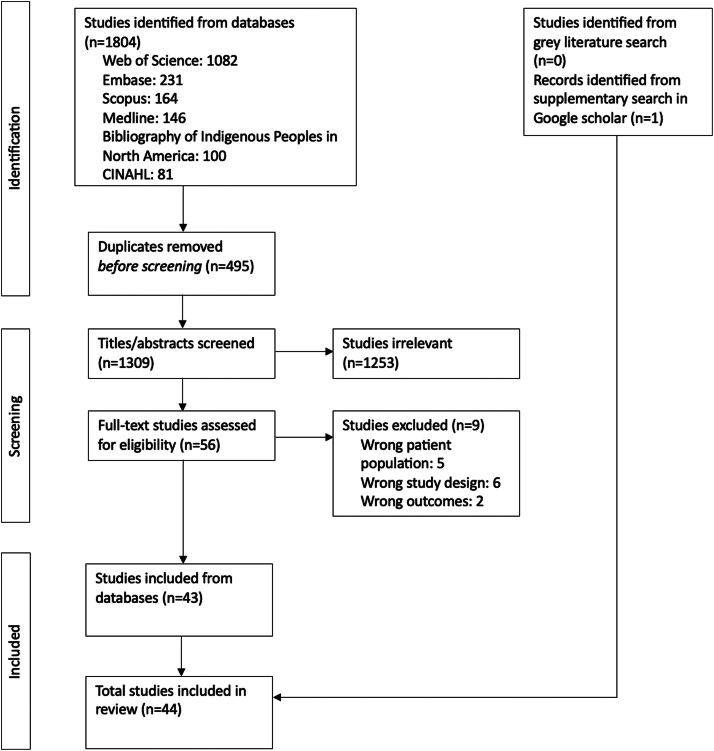
Flow chart of articles identification, screening, and inclusion process.

**Figure 2:**
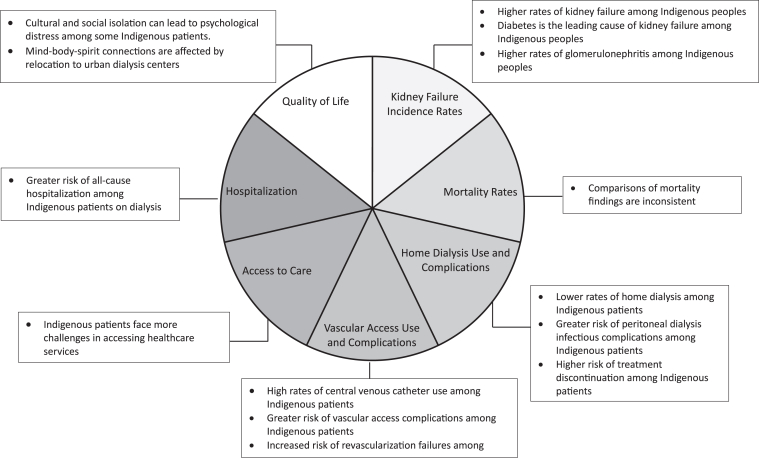
Summarized key findings.

Forty-four (44) studies met the inclusion criteria for the scoping review (Fig 1, Table 1).^23^^,^^25^, ^26^, ^27^, ^28^, ^29^, ^30^, ^31^, ^32^, ^33^, ^34^, ^35^, ^36^, ^37^, ^38^, ^39^, ^40^, ^41^, ^42^, ^43^, ^44^, ^45^, ^46^, ^47^, ^48^, ^49^, ^50^, ^51^, ^52^, ^53^, ^54^, ^55^, ^56^, ^57^, ^58^, ^59^, ^60^, ^61^, ^62^, ^63^, ^64^, ^65^, ^66^, ^67^ The majority of articles were retrospective quantitative studies (32/44, 72.7%).^25^^,^^27^, ^28^, ^29^^,^^34^, ^35^, ^36^, ^37^, ^38^, ^39^, ^40^, ^41^, ^42^, ^43^, ^44^, ^45^, ^46^^,^^48^, ^49^, ^50^, ^51^, ^52^, ^53^, ^54^, ^55^, ^56^, ^57^, ^58^, ^59^, ^60^^,^^63^^,^^64^ The rest consisted of 5 prospective quantitative studies,^23^^,^^26^^,^^30^^,^^32^^,^^61^ 3 qualitative retrospective studies,^31^^,^^33^^,^^65^ 2 qualitative cross-sectional studies,^66^^,^^67^ 1 quantitative cross-sectional study,^62^ and 1 case study.^47^ The sample size of the articles varied between 13 and 1,816,824 participants. In total, 81.8% (36/44) of articles were published in the last 20 years (2004-2021), of which 6 were published in the last 5 years (≥2019). Studies were conducted across Canada (15/44, 34.1%); in Saskatchewan (9/44, 20.5%), in Alberta, Saskatchewan and Manitoba (2/44, 4.5%); in Ontario and/or James Bay (7/44, 15.9%); in Manitoba (8/44, 18.2%); in Ontario and Manitoba (1/44, 2.3%); in Quebec (1/44, 2.3%); and internationally including Canada (1/44, 2.3%). Two studies (4.5%) specifically included Cree communities, 15 (34.1%) focused on First Nations, 2 (4.5%) addressed Metis populations, and the remaining 25 studies (56.8%) did not specify the included Indigenous communities. Seven non-mutually exclusive outcome categories were created. Articles were classified within 1 or more of these categories as such: kidney failure incidence rates (19/44, 43.2%), mortality rates (8/44, 18.2%), home dialysis use and complications (13/44, 29.5%), vascular access use and complications (6/44, 13.6%), access to care (6/44, 13.6%), hospitalization (2/44, 4.5%) and quality of life (7/44, 15.9%). Key findings of the included studies are summarized in Fig 2.

**Table 1:** Summary of Extracted Data of Included Articles

Authors	Title	Year	Location	Indigenous Communities	Study Design	Sample Size	Aim	Key Findings	Results Categories
Young et al^28^	Excessive burden of end-stage renal disease among Canadian Indians: a national survey	1989	Canada	N/A	Quantitative, retrospective	8,432 kidney failure patients: 304 Indigenous and 8,128 non-Indigenous	Assess multiple aspects of kidney failure within Canadian Indigenous communities.	The incidence rate of kidney failure among Indigenous individuals was up to 4 times the Canadian rate. More cases were due to diabetes and glomerulonephritis in Indigenous patients.	1. Kidney failure incidence rates
Wilson et al^29^	Incidence and prevalence of end-stage renal disease among Ontario's James Bay Cree	1992	James Bay	Cree	Quantitative, retrospective	38 patients: 10 kidney failure Indigenous patients and 28 chronic kidney disease patients	Compare the incidence rates of kidney failure between the James Bay Cree community and non-Indigenous individuals in Ontario.	Kidney failure rates were 3.2 times higher in 1989 among the James Bay Cree community than non-Indigenous individuals, but they were lower than the average rate of all Indigenous Canadians.	1. Kidney failure incidence rates
Dyck et al^27^	Rates and outcomes of diabetic end-stage renal disease among registered native people in Saskatchewan	1994	Saskatchewan	N/A	Quantitative, retrospective	645 kidney failure patients: 89 Indigenous and 556 non-Indigenous	Compare the rates and outcomes of diabetic kidney failure between Indigenous and non-Indigenous people.	Rates of diabetic kidney failure were greater in every age group among Indigenous individuals than in non-Indigenous individuals, with an overall relative risk ratio of 16.2. Indigenous patients with diabetes were 7 times more likely than non-Indigenous diabetics to have diabetic kidney failure.	1. Kidney failure incidence rates
Fine et al^30^	Higher incidence of peritonitis in native Canadians on continuous ambulatory peritoneal dialysis	1994	Canada	N/A	Quantitative, prospective	184 dialysis patients: 48 Indigenous and 136 non-Indigenous	Compare rates of peritoneal dialysis between Indigenous and non-Indigenous Canadians.	The risk of developing peritonitis after 6 months of starting home dialysis was 2.5 times higher among Indigenous Canadians than non-Indigenous Canadians. The risk of exit-site infection was 2.2 times higher among Indigenous Canadians than non-Indigenous Canadians.	1. Home dialysis use and complications
Wilson et al^31^	Native Canadians relocating for renal dialysis. Psychosocial and cultural issues	1994	James Bay	N/A	Qualitative, retrospective	24 dialysis Indigenous patients	Investigate the impact of relocating Indigenous patients for dialysis treatment.	Relocating for dialysis disrupted social support systems and gave increase to psychosocial issues. Cost, equipment availability, and training of health care workers made remote care more challenging.	1. Quality of life
Bulloch et al^32^	Excess prevalence of non-diabetic renal disease in native American children in Manitoba	1996	Manitoba	First Nations	Quantitative, prospective	247 kidney failure patients: 73 Indigenous and 174 non-Indigenous	Confirm the increased prevalence of nondiabetic kidney disease among Indigenous patients of Manitoba.	Indigenous children in Manitoba were 4.5 times more likely to develop congenital kidney disease and 6.1 times more likely to develop acquired kidney disease than non-Indigenous children and were at higher risk of chronic kidney failure. A higher proportion of Indigenous children than non-Indigenous children had difficulty accessing medical services and traveled longer distances to receive treatments.	1. Kidney failure incidence rates 2. Access to care
Dyck et al^33^	Non-diabetic end-stage renal disease among Saskatchewan aboriginal people	1998	Saskatchewan		Qualitative, retrospective	645 kidney failure patients: 89 Indigenous and 556 non-Indigenous	Compare the rates, causes and outcomes of nondiabetic kidney failure between Indigenous and non-Indigenous Canadians.	Diabetes was the main contributor to kidney failure among the Indigenous population, accounting for 56% of cases. Indigenous peoples had a 2.5-fold increase in the incidence of nondiabetic kidney failure compared with the non-Indigenous population.	1. Kidney failure incidence rates
Davidson et al^34^	Steal syndrome complicating upper extremity hemoaccess procedures: incidence and risk factors	2003	Manitoba	N/A	Quantitative, retrospective	325 procedures (217 dialysis patients): 40 Indigenous and 285 non-Indigenous	Investigate the incidence rates of vascular steal syndrome in dialysis patients with upper extremity hemoaccess and.	Indigenous dialysis patients with were at 2.6 times higher risk of vascular steal syndrome in upper extremity hemoaccess procedures.	1. Vascular access use and complications
Tonelli et al^26^	Death and renal transplantation among Aboriginal people undergoing dialysis	2004	Alberta, Saskatchewan, and Manitoba	N/A	Quantitative, prospective	4,333 dialysis patients: 685 Indigenous and 3,648 non-Indigenous	Compare differences in survival between Indigenous and non-Indigenous patients.	Adjusted survival rates for dialysis patients were similar for Indigenous and non-Indigenous patients (adjusted hazards ratio of 0.89).	1. Mortality rates
Tonelli et al^35^	Use and outcomes of peritoneal dialysis among Aboriginal people in Canada	2005	Alberta, Saskatchewan, and Manitoba	N/A	Quantitative, retrospective	3,823 dialysis patients: 685 Indigenous and 3,138 non-Indigenous	Determine home dialysis rates, technique failure and mortality outcomes in Indigenous dialysis patients.	Home dialysis was 49% less frequent in Indigenous patients compared with non-Indigenous patients. Indigenous patients were 46% more likely to deal with technique failure, but the risk of death was similar between both groups.	1. Mortality rates 2. Home dialysis use and complications
Chou et al^36^	Quality of care among Aboriginal hemodialysis patients	2006	Canada	N/A	Quantitative, retrospective	835 dialysis patients: 95 Indigenous and 740 non-Indigenous	Compare the quality of hemodialysis between Indigenous and non-Indigenous Canadians.	There was similar quality of hemodialysis care for Indigenous and non-Indigenous patients. However, Indigenous patients were 1.5 times less likely to achieve predialysis blood pressure control and 1.4-1.7 times less likely to achieve mineral metabolism.	1. Vascular access use and complications 2. Access to care 3. Quality of life
Goulet et al^37^	Revascularization for peripheral vascular disease in Aboriginal and non-Aboriginal patients	2006	Canada	N/A	Quantitative, retrospective	678 peripheral vascular disease patients: 84 Indigenous and 594 non-Indigenous	Examine the outcomes following arterial bypass surgery between Indigenous and non-Indigenous patients.	Indigenous patients had worse revascularization outcomes than non-Indigenous patients. For example, 58% of Indigenous patients developed gangrene as a complication from the surgery while 16% of non-Indigenous patients developed this complication.	1. Vascular access use and complications
Iliescu et al^38^	Modality choice among Aboriginal incident dialysis patients--influence of geographic location	2006	Ontario and James Bay	N/A	Quantitative, retrospective	39 dialysis Indigenous patients	Evaluate the number of Indigenous patients who chose home dialysis and identify the reasons for their choice of modality.	Indigenous patients chose home dialysis because of limited modality options. Home dialysis was available in both studied regions, while hemodialysis only in Southeastern Ontario.	1. Home dialysis use and complications
Stewart et al^39^	The enigma of hypertensive ESRD: observations on incidence and trends in 18 European, Canadian, and Asian-Pacific populations, 1998 to 2002	2006	International including Canada	N/A	Quantitative, retrospective	N/A	Determine the correct hypertensive kidney failure rates among Indigenous and non-Indigenous populations from many countries.	The rate of hypertensive kidney failure was 46/million in Indigenous Canadians compared with 50 million in non-Indigenous Canadians. The difference between both populations was nonsignificant.	1. Kidney failure incidence rates
Gao et al^40^	Prevalence of chronic kidney disease and survival among aboriginal people	2007	Canada	N/A	Quantitative, retrospective	673 653 individuals: 14 989 Indigenous and 658 664 non-Indigenous	Examine the occurrence of kidney failure and the survival rates with the disease within the Indigenous population.	The incidence of kidney failure was approximately twice as high among Indigenous patients than non-Indigenous patients. Indigenous patients also had a 77% higher adjusted risk of mortality compared with non-Indigenous patients.	1. Kidney failure incidence rates 2. Mortality rates
McIntyre et al^41^	Foot and ankle problems of Aboriginal and non-Aboriginal diabetic patients with end-stage renal disease	2007	Canada	N/A	Quantitative, retrospective	77 dialysis patients: 36 Indigenous and 41 non-Indigenous	Compare rates of lower extremity complications in diabetic kidney failure patients in Indigenous and non-Indigenous populations.	Lower extremity complications were more prevalent among diabetic kidney failure Indigenous patients. For example, incidence of lower extremity amputation was 36% among Indigenous patients while it was 17% among non-Indigenous patients (*P* value < 0.05).	1. Vascular access use and complications outcomes 2. Quality of life
Gao et al^42^	Access to health care among status Aboriginal people with chronic kidney disease	2008	Canada	N/A	Quantitative, retrospective	107.693 dialysis patients: 1,182 Indigenous and 106, 511 non-Indigenous	Compare the access to care between Indigenous and non-Indigenous dialysis patients.	Dialysis Indigenous patients were twice more likely to be hospitalized for conditions that could have been managed as outpatient and were 43% less likely to have access to nephrology appointments.	1. Access to care
Dyck et al^43^	Differences in glycemic control and survival predict higher ESRD rates in diabetic First Nations adults	2010	Saskatchewan	First Nations	Quantitative, retrospective	24,207 diabetic patients: 2,321 Indigenous and 21,886 non-Indigenous	Examine why diabetic kidney failure is more prevalent among First Nations individuals than non-First Nations individuals.	Indigenous diabetic patients experienced worse glycemic control than non-Indigenous patients at every level of chronic kidney disease with a mean glycated hemoglobin of 8.16% among Indigenous patients compared with 7.36% among non-Indigenous patients.	1. Kidney failure incidence rates
Hildebrand et al^44^	Peritonitis and exit site infections in First Nations patients on peritoneal dialysis	2010	Manitoba	First Nations	Quantitative, retrospective	727 dialysis patients: 161 Indigenous and 566 non-Indigenous	Compare rates of peritonitis and exit-site infections between Indigenous and non-Indigenous patients on peritoneal dialysis.	Indigenous patients had higher rates of peritonitis but similar rates of exit-site infections than non-Indigenous patients (86.0/100 patient-years among Indigenous patients vs 78.2/100 patient-years among non-Indigenous patients).	1. Home dialysis use and complications
Lafrance et al^45^	Vascular access-related bloodstream infections in First Nations, community and teaching Canadian dialysis units, and other centre-level predictors	2010	Quebec	First Nations	Quantitative, retrospective	621 dialysis Indigenous patients	Examine if there is a variability in bacteremia risk between different dialysis centers deserving Indigenous patients.	There was a high number of dialysis Indigenous patients who were using central venous catheters for dialysis (76.7% of patients were initiated with a central venous catheter). There was no difference in bacteremia risk between the studied dialysis centers.	1. Vascular access use and complications
Sood et al^46^	Adverse outcomes among Aboriginal patients receiving peritoneal dialysis	2010	Manitoba	N/A	Quantitative, retrospective	727 dialysis: 161 Indigenous and 631 non-Indigenous	Investigate the incidence of mortality, technique failure, and peritonitis in Indigenous patients on home dialysis.	Indigenous patients on peritoneal dialysis had a 48% higher risk of mortality and had a 79% higher risk of peritonitis than non-Indigenous patients.	1. Mortality rates 2. Home dialysis use and complications
Kolewaski et al^47^	Relocating from the Mushkegowuk Territory for Hemodialysis: The Cree Illness Experience and Perceived Quality of Life	2010	Ontario	Cree	Qualitative, case study	13 participants: 4 Cree hemodialysis patients, 3 nephrologists, 3 nurses and 3 Weeneebayko Patient Service workers	Define the experience of Cree patients who relocated to receive hemodialysis and how it has affected their quality of life.	Cultural shock from relocation was felt by study participants. Patients found their mind–body–spirit connection to be affected.	1. Quality of life
Dyck et al^48^	End Stage Renal Disease Among People with Diabetes: A Comparison of First Nations People and Other Saskatchewan Residents from 1981 to 2005	2010	Saskatchewan	First Nations	Quantitative, retrospective	1,226 kidney failure patients: 320 Indigenous and 816 non-Indigenous	Compare the prevalence and incidence of kidney failure between Indigenous and non-Indigenous patients with diabetes in Saskatchewan.	Kidney failure incidence among Indigenous with diabetes was 3 to 4 times higher than non-Indigenous between 1991 and 1996. On average, it took longer for Indigenous patients with diabetes to develop kidney failure, but their survival rate was lower than non-Indigenous patients.	1. Kidney failure incidence rates 2. Mortality rates
Martens et al^49^	What is the comparative health status and associated risk factors for the Metis? A population-based study in Manitoba, Canada	2011	Manitoba	Metis	Quantitative, retrospective	1 177 688 individuals: 73 016 Indigenous and 1 104 672 non-Indigenous	Compare the health status between Metis and non-Metis residents of Manitoba.	Metis living in Manitoba had higher all-cause mortality (9.7/1000 vs 8.4/1000) and morbidity rates, such as diabetes (29% more likely) than non-Metis individuals. The rate of dialysis initiation was 35 % higher for Metis people than non-Metis residents of Manitoba.	1. Kidney failure incidence rates
Samuel et al^50^	Incidence and causes of end-stage renal disease among Aboriginal children and young adults	2012	Canada	N/A	Quantitative, retrospective	980 young individuals (age < 22 y): 159 Indigenous and 821 non-Indigenous	Compare kidney failure incidence rates between Indigenous and non-Indigenous children and young adults in Canada.	Indigenous children and young adults were 2.18 more likely to have glomerulonephritis than their non-Indigenous counterpart.	1. Kidney failure incidence rates
Sood et al^51^	Association of modality with mortality among Canadian Aboriginals	2012	Canada	N/A	Quantitative, retrospective	31.576 dialysis patients: 2,393 Indigenous and 29,183 non-Indigenous	Evaluate whether dialysis modality affects mortality in Indigenous dialysis patients.	Indigenous patients on home dialysis experienced a higher mortality rate (adjusted hazards ratio of 1.36) and a higher incidence of technique failure (adjusted hazards ratio of 1.29) in comparison to Whites.	1. Mortality rates 2. Home dialysis use and complications
Canadian Institute for Health Information^52^	End-Stage Renal Disease Among Aboriginal Peoples in Canada: Treatment and Outcomes	2013	Canada	N/A	Quantitative, retrospective	N/A	Investigate the factors causing differences in kidney failure outcomes between Indigenous and non-Indigenous patients.	Indigenous kidney failure patients were younger (median age almost a decade younger). Most patients lived in remote regions and traveled longer distances for dialysis (20% of Indigenous patients traveled more than 250 km vs 5% of non-Indigenous patients). They also had a higher adjusted mortality rate (hazards ratio of 1.33 after controlling for age).	1. Kidney failure incidence rates 2. Mortality rates 3. Quality of life
Nessim et al^53^	Frequency and microbiology of peritonitis and exit-site infection among obese peritoneal dialysis patients	2013	Manitoba	N/A	Quantitative, retrospective	938 dialysis patients: 220 Indigenous and 718 non-Indigenous	Determine the frequency of peritonitis and exit-site infection among obese home dialysis patients.	There was no correlation between obesity and an elevated risk of peritonitis in Canadians on home dialysis.	1. Home dialysis use and complications
Dyck et al^54^	The long-term risks of end stage renal disease and mortality among First Nations and non-First Nations people with youth-onset diabetes	2014	Saskatchewan		Quantitative, retrospective	2,640 kidney failure patients: 352 Indigenous and 2,288 non-Indigenous	Examine the risk of kidney failure incidence and mortality in patients with youth-onset diabetes.	Indigenous individuals with youth-onset diabetes had 2.59 higher risks of kidney failure and 2.64 higher risk of mortality than non-Indigenous patients.	1. Kidney failure incidence rates
Jiang et al^55^	Differential mortality and the excess burden of end-stage renal disease among First Nations people with diabetes mellitus: a competing-risks analysis	2014	Saskatchewan	First Nations	Quantitative, retrospective	90,429 diabetic patients: 8,254 Indigenous and 82 175 non-Indigenous	Investigate the reasons for differences in diabetic kidney failure mortality rates between Indigenous and non-Indigenous patients.	Indigenous patients with diabetes were at 2.66 times higher risk of kidney failure and were younger when diagnosed with diabetes, causing a higher risk of developing kidney failure.	1. Kidney failure incidence rates
Samuel et al^56^	Association between First Nations ethnicity and progression to kidney failure by presence and severity of albuminuria	2014	Canada	First Nations	Quantitative, retrospective	1,816,824 individuals: 48,669 Indigenous and 1,768,155 non-Indigenous	Evaluate if albuminuria plays a role in the progression from to kidney failure among Indigenous patients.	Indigenous and non-Indigenous individuals had a similar risk of developing kidney failure when presenting with albuminuria.	1. Kidney failure incidence rates
Canadian Institute for Health Information^57^	High Risk and High Cost: Focus on Opportunities to Reduce Hospitalizations of Dialysis Patients in Canada	2016	Canada	N/A	Quantitative, retrospective	38,369 dialysis patient: 1,777 Indigenous and 36,592 non-Indigenous	Evaluate which are the primary factors accounting for hospitalizations among patients on dialysis in Canada.	Indigenous patients were 30% more at risk of hospitalization from dialysis-related infections than non-Indigenous. They were also 20% more likely of all-cause hospitalizations.	1. Hospitalization
Hayward et al^58^	Kidney Disease Among Registered Metis Citizens of Ontario: A Population-Based Cohort Study	2017	Ontario	Metis	Quantitative, retrospective	61,145 individuals: 12,229 Indigenous and 48 916 non-Indigenous	Compare the rates of kidney failure between Metis and non-Metis residents of Ontario.	Kidney failure rates were slightly higher for Metis living in Ontario but were overall very similar (<3.0 per 10,000 person-years, 0 = 0.73). .	1. Kidney failure incidence rates
Trinh et al^25^	Racial Differences in Home Dialysis Utilization and Outcomes in Canada	2017	Canada	N/A	Quantitative, retrospective	66 600 dialysis patients: 3,866 Indigenous and 62 734 non-Indigenous	Compare home dialysis rates between Indigenous and non-Indigenous dialysis patients.	Indigenous dialysis patients had more comorbid conditions and a 29% reduced likelihood of being on home dialysis than non-Indigenous Canadians. They were also at 19% increased risk of technique failure and 16% increased risk of death from home dialysis.	1. Mortality rates 2. Home dialysis use and complications
Molnar et al^59^	Hospitalizations in Dialysis Patients in Canada: A National Cohort	2018	Provinces and territories across Canada (excluding Manitoba and Quebec)	N/A	Quantitative, retrospective	38 369 dialysis patients: 1,777 Indigenous and 36 592 non-Indigenous	Compare the risk of hospitalization between Indigenous and non-Indigenous dialysis patients.	Indigenous patients were 20% more likely of all-cause hospitalization following dialysis start.	1. Hospitalization 2. Quality of life
Thomas et al^60^	A Retrospective Study of Chronic Kidney Disease Burden in Saskatchewan's First Nations People	2018	Saskatchewan	First Nations	Quantitative, retrospective	2,478 chronic kidney disease patients: 379 Indigenous and 2,099 non-Indigenous	Determine the extent of chronic kidney disease severity and the access health care for Indigenous patients living in Saskatchewan.	Indigenous patients with chronic kidney disease had higher rates of kidney failure than non-Indigenous patients (33.0% vs 21.4%) and had lower rates of home dialysis use than non-Indigenous patients (16.2% vs 25.7%). They also needed to travel longer distances to access health care.	1. Home dialysis use and complications 2. Quality of life
Mathew et al^61^	Barriers to Peritoneal Dialysis in Aboriginal Patients	2018	Ontario and Manitoba	First Nations	Quantitative, prospective	99 dialysis patients: 32 Indigenous and 67 non-Indigenous	Evaluate barriers to home dialysis use among Indigenous dialysis patients.	The main barriers to home dialysis for Indigenous patients were anxiety and economic factors.	1. Home dialysis use and complications
Harasemiw et al^62^	Remote Dwelling Location Is a Risk Factor for CKD Among Indigenous Canadians	2018	Manitoba	First Nations	Quantitative, cross-sectional	1,630 Indigenous participants	Evaluate the factors that lead to higher rates of kidney failure in Canadian Indigenous communities.	Indigenous individuals living in remote areas had a 1.9- to 3.3-fold higher risk of kidney failure compared with Indigenous populations in urban and rural settings.	1. Access to care
Kelly et al^63^	Prevalence of chronic kidney disease and cardiovascular comorbid conditions in adults in First Nations communities in northwest Ontario: a retrospective observational study	2019	Ontario	First Nations	Quantitative, retrospective	16,170 Indigenous individuals	Identify the prevalence of diabetes, hypertension and poor lipid profiles in Indigenous chronic kidney disease patients living in Ontario.	The prevalence of advanced chronic kidney disease in the Indigenous population living in Ontario was 7%. 72% of Indigenous patients with chronic kidney disease also had diabetes and 71% dyslipidemia, which increases their risk of cardiovascular diseases.	1. Kidney failure incidence rates 2. Vascular access use and complications
Nash et al^64^	Kidney disease and care among First Nations people with diabetes in Ontario: a population-based cohort study	2019	Canada	First Nations	Quantitative, retrospective	21,968 diabetic Indigenous patients	Compare the incidence and prevalence of kidney failure between Indigenous and non-Indigenous diabetic patients.	Indigenous patients with diabetes had a 2.9% higher incidence rate of kidney failure than non-Indigenous patients, but the quality of care was the same between both patient populations.	1. Kidney failure incidence rates 2. Access to care
Lavoie et al^65^	Is Assisted Peritoneal Dialysis a Solution for Northern Manitoba?	2019	Manitoba	First Nations	Qualitative, retrospective	55 participants: 14 Indigenous dialysis patients, 14 Indigenous family caregivers and 27 health care providers and administrators	Understand the experience of Indigenous patients and their family members with home dialysis.	The main barriers to home dialysis were medical suitability, lack of trust and fear of complications. Lack of support from peers and inappropriate housing were other barriers.	1. Home dialysis use and complications
Prasad et al^66^	Barriers to Peritoneal Dialysis in Saskatchewan Canada: Results From a Province-Wide Survey	2020	Saskatchewan	First Nations	Qualitative, cross-sectional	366 dialysis patients: 267 Indigenous and 99 non-Indigenous	Evaluate the main barriers to home dialysis faced by Indigenous patients living on reserves vs off reserves.	Indigenous patients said being satisfied with their treatments and being reluctant to home dialysis. They associated home dialysis with higher rates of infections and with lower success rates than hemodialysis.	1. Home dialysis use and complications
Richels et al^67^	Community-Based Dialysis in Saskatchewan First Nations: A Grassroots Approach to Gaining Insight and Perspective From First Nations Patients With Chronic Kidney Disease	2020	Saskatchewan	First Nations	Qualitative, cross-sectional	71 Indigenous patients	Identify the challenges to home dialysis among Indigenous communities.	Multiple challenges to home dialysis among Indigenous patients were identified. These included education on kidney failure, proper and culturally safe training, home dialysis safety and support from community members.	1. Home dialysis use and complications
Domonkos et al^23^	Characteristics of End-Stage Kidney Disease in a Cohort of Indigenous and Non-Indigenous Adults in Northwestern Ontario, Canada	2021	Ontario	N/A	Quantitative, prospective	185 kidney failure patients: 91 Indigenous and 94 non-Indigenous	Compare epidemiologic and clinical outcomes between Indigenous and non-Indigenous patients on dialysis.	Indigenous patients with kidney failure were younger at time of disease onset and the severity of their disease was higher. Diabetes was the most common etiology for the disease and was more prevalent among Indigenous patients (52.7% of Indigenous patients vs 37.2% of non-Indigenous patients). IgA nephropathy was also more common among Indigenous patients (9.9% of Indigenous patients vs 1.1% of non-Indigenous patients). Pneumococcal immunization rate was the lowest among Indigenous patients on dialysis (33% of Indigenous patients vs 52.1% of non-Indigenous patients).	1. Kidney failure incidence rates 2. Access to care

### Kidney Failure Incidence Rates

#### Higher Rates of Kidney Failure Among Indigenous Patients

Fifteen studies consistently demonstrated higher rates of kidney failure among Indigenous peoples in Canada compared with non-Indigenous peoples, with rates ranging from 1.35 to 4 times higher.^23^^,^^27^, ^28^, ^29^^,^^32^^,^^33^^,^^39^^,^^40^^,^^48^, ^49^, ^50^^,^^54^, ^55^, ^56^^,^^64^ A cohort study from Young et al^28^ of 8,432 kidney failure patients across Canada, including 304 patients Indigenous patients, demonstrated that Indigenous individuals were 2.5 to 4 times more likely to develop kidney failure than non-Indigenous Canadians. Additionally, Gao et al^40^ noted a 2-fold increased rate of severe chronic kidney disease (CKD) among First Nations patients of Alberta compared with non-First Nations patients within the same province. The incidence rate of kidney failure among the James Bay Cree community in Northern Ontario was found to be 1.8 times higher than that of non-Indigenous Canadians.^29^ For their part, Metis residents of Manitoba initiated dialysis 35% more often than non-Metis residents of the same province.^49^ Conversely, Metis living in Ontario experienced almost the same rate of dialysis start than non-Metis Ontarians.^58^ First Nations children also experienced higher rates of kidney failure.^32^ Indeed, First Nations children of Manitoba were 6.3 times more likely to develop CKD, with higher prevalence of congenital and acquired kidney diseases than all non-Indigenous children.^32^

#### Diabetes as the Leading Cause of Kidney Failure Among Indigenous Patients

Diabetes was consistently the leading cause of kidney failure among Indigenous peoples.^23^^,^^27^^,^^28^^,^^43^^,^^54^^,^^55^^,^^64^ Domonkos et al^23^ reported that 52.7% of kidney failure cases among Indigenous patients were due to diabetes, compared with 37.2% of cases among non-Indigenous participants. Indigenous patients with diabetes experienced worse glycemic control at every stage of CKD and were 7 times more likely to develop kidney failure than non-Indigenous patients with diabetes.^27^ This increased risk of developing kidney failure from diabetes among Indigenous patients was seen to persist across all age groups.^27^^,^^43^

#### Increased Rates of Nondiabetic Kidney Failure Among Indigenous Patients

Canadian Indigenous patients were 2.5 times more likely to develop non-diabetic kidney failure than all non-Indigenous Canadians.^33^ Indeed, rates of glomerulonephritis, including IgA nephropathy, proliferative nephropathies and poststreptococcal glomerulonephritis, were disproportionally higher among Indigenous adults and children, even when accounting for comorbid conditions.^23^^,^^28^^,^^33^^,^^50^

### Mortality Rates

#### Inconsistent Findings on Dialysis Mortality Rates

Comparisons of dialysis mortality rates between Indigenous and non-Indigenous patients were inconsistent. For instance, 2 studies comparing White and Indigenous dialysis patients from multiple Canadian provinces found no difference in mortality rates between both patient populations, regardless of dialysis modality.^26^^,^^35^ In contrast, a study by Gao et al^40^ demonstrated that Indigenous dialysis patients were at 77% higher risk of mortality compared with non-Indigenous patients. The Canadian Institute for Health Information published similar findings, indicating that Indigenous dialysis patients had a 33% increased risk of mortality compared with non-Indigenous patients after adjusting for age.^52^ Finally, Dyck et al^48^ found a shorter median survival at 2 years among dialysis Indigenous patients with diabetes than non-Indigenous patients with diabetes, even if younger at the time of diagnosis.

#### Higher Rates of Peritoneal Dialysis Mortality among Indigenous Patients

Results from 3 studies showed a 16% to 48% higher peritoneal dialysis mortality risk among Indigenous patients compared with non-Indigenous counterparts.^25^^,^^46^^,^^51^

### Home Dialysis Use and Complications

#### Decreased Use of Home Dialysis Among Indigenous Patients in Most Canadian Regions

Another consistent finding was the lower rates of home dialysis uptake among Canadian Indigenous patients compared with non-Indigenous patients; this was reported in 3 different studies.^25^^,^^35^^,^^60^ For example, in a Canadian-wide retrospective study using data from the Canadian Organ Replacement Register, Trinh et al^25^ reported a significantly lower use of home dialysis among Indigenous patients with an adjusted odds ratio of 0.71 compared with White patients. Interestingly, a study by Iliescu et al^38^ reported a higher rate of home dialysis use among Indigenous patients living in the Northern Ontario James Bay Coastal area, equal to 48.7%. However, the study’s authors noted that hemodialysis was not an option in this region, which may have affected its findings.^38^ Exceptionally, one prospective cohort study from Mathew et al^61^ composed of 99 dialysis patients from Ontario and Manitoba, of whom 32 were Indigenous, recorded no significant difference in the use of home dialysis between Indigenous and non-Indigenous patients.

#### Higher Rates of Home Dialysis Complications Among Indigenous Patients

Studies demonstrated differences in home dialysis complications rates among Indigenous patients.^25^^,^^30^^,^^35^^,^^44^^,^^46^^,^^51^ Three studies showed an increased risk of peritonitis among Indigenous patients compared with other Canadians,^30^^,^^44^^,^^46^ one of which also reported an increased risk of exit-site infections.^30^ Sood et al^46^ found a 2.24 to 2.59 times higher risk of peritonitis among Indigenous patients of Manitoba compared with all non-Indigenous patients. Similarly, Hildebrand et al^44^ showed the total peritonitis rate was of 132.7/100 patient-years in First Nations patients versus 87.8/100 patient-years in all non-First Nations patients. Three studies found that Indigenous patients faced 1.19 to 1.46 times higher rates of home dialysis discontinuation because of increased infection rates, compared with White patients.^25^^,^^35^^,^^51^

#### Barriers to Home Dialysis Among Indigenous Patients

The main identified barriers to home dialysis use for Indigenous patients were anxiety, financial constraints, burden to family members, fear of infections, and fear of home dialysis resulting in inferior care.^61^^,^^65^, ^66^, ^67^ Ten patients from a study conducted by Prasad et al^66^ believed the transition from in-center hemodialysis to home dialysis was an effort by the government to oppress Indigenous patients and decrease their life span. Richels et al^67^ put emphasis on providing culturally safe training and education on kidney failure and on home dialysis to overcome these challenges.

### Hospitalization

#### Higher Rates of Hospitalization Among Indigenous Patients

In a large Canadian retrospective study using data from the Canadian Organ Replacement Register, Molnar et al^59^ found a 1.20 times higher risk of all-cause hospitalization following dialysis start among Indigenous peoples when compared with White Canadians. The Canadian Institute for Health Information also reported higher rates of hospitalizations among Indigenous patients, who were found to be 1.3 times more likely to be hospitalized from dialysis infections and 1.2 times from all other causes.^57^

### Vascular Access Use and Complications

#### High rates of Central Venous Catheter Use Among Indigenous Patients

In a study by Lafrance et al^45^ conducted in 7 outpatient dialysis centers across Quebec, central venous catheter use among First Nations patients was found to be as high as 70%. Despite this unfavorable quality of care indicator, the same study demonstrated similar incidence rates of bacteremia when comparing First Nations and non-First Nations patients treated at different dialysis centers.^45^

#### Higher Rates of Vascular Complications Among Indigenous Patients

Davidson et al^34^ reported that Indigenous patients were 3.59 times more likely to suffer from vascular steal syndrome as a complication of their upper extremity vascular access procedure than all non-Indigenous patients. The authors hypothesized that it might be related to other prevalent comorbid conditions present within this patient population, specifically peripheral vascular disease.^34^ Moreover, a multivariate analysis conducted by Goulet et al^37^ reported that Indigenous patients with CKD exhibit poorer outcomes after undergoing revascularization surgery compared with non-Indigenous patients, including higher rates of unsuccessful limb salvage (18% vs 9%) and increased loss of blood flow within grafts (36% vs 22%).

### Access to Care

#### Inconsistent Findings on Access to Care and Quality of Care

Three studies demonstrated that Canadian Indigenous patients face challenges with access to health care services.^32^^,^^42^^,^^62^ Gao et al^42^ observed that kidney failure Indigenous patients were 43% less likely than non-Indigenous patients to obtain a nephrology appointment. Another study from Domonkos et al^23^ showed that Indigenous patients had the lowest rate of vaccination against pneumococcal infection, even if most of the Indigenous participants lived in urban settings. Conversely, Nash et al^64^ recently published a study indicating that First Nations patients with kidney failure and diabetes living in Ontario had equal access to nephrology care postreferral than non-Indigenous Ontarians. However, this same study reported a lower amount of follow-up laboratory tests for kidney health monitoring and therapy adjustment among Indigenous patients compared with non-Indigenous Ontarians.^64^ Chou et al^36^ also showed equal quality of hemodialysis treatments for both, Indigenous and non-Indigenous inhabitants of Alberta, except for a higher incidence of suboptimal predialysis clinical metrics among Indigenous patients.

### Quality of Life

#### Longer Travel Distances to Health Care Centers for Indigenous Patients

Four studies assessed quality of life measures among Indigenous patients through quantitative and qualitative data, although none used validated scores.^31^^,^^47^^,^^52^^,^^60^ Long travel time for access to health care was identified as a major challenge affecting quality of life for Indigenous dialysis patients.^52^^,^^60^ Although 80% of non-Indigenous patients lived within 50 km of their dialysis clinic, this was true for only 53% of Indigenous patients.^52^ Furthermore, 20% of Indigenous dialysis patients traveled more than 250 km for health care services, whereas this was the case for only 5% of non-Indigenous Canadians.^52^ Additionally, Indigenous patients who decided to relocate and live closer to their dialysis unit were confronted with disruption of their social support systems, which may further impair many aspects of their quality of life.^31^

#### Burdens of Relocation for Closer Access to Health Care Services

Wilson et al^31^ conducted interviews with 24 residents of the Moose Factory Zone in James Bay Ontario, living on and off reserves, who were either CKD patients, community spokespersons, or caregivers. Participants reported that the current relocation system to access kidney treatments lacks social support, disrupts family roles, and imposes financial burdens. Participants acknowledged that having a local dialysis center would alleviate their current quality of life concerns, but they also recalled the importance of ensuring high quality care if local centers are established.^31^ Kolewasky et al^47^ also conducted interviews with 4 Cree hemodialysis patients who had left the Muskegowuk Territory to obtain urban hemodialysis treatments. All Cree patients explained having experienced many difficult challenges from relocation, including cultural shock leading to isolation. Leaving their family to receive treatment was noted to be the most challenging aspect of relocation by all patients. Cultural and social isolation resulted in significant psychological disturbances for some patients, including depression. Furthermore, Cree patients found their mind–body–spirit connections to be affected by the relocation, and felt misunderstood by health care providers, further affecting their overall quality of life. Lastly, participants of this study acknowledged that frequent visits to their community helped improve their overall well-being.^47^

## Discussion

This scoping review summarized results from 44 studies and highlighted significant disparities in clinical outcomes between Indigenous and non-Indigenous patients treated with dialysis in Canada. Indigenous peoples in Canada face a higher prevalence of kidney failure, more frequent dialysis-related complications, higher rates of hospitalization, lower rates of arteriovenous fistulas, lower use of home dialysis, reduced access to health care, and poorer quality of life. However, data reporting mortality differences was inconsistent.

Despite a public health care model, research has demonstrated significant health inequities affecting Indigenous peoples.^1^ These disparities are rooted in a complex interplay of factors, including fragmented and limited access to health care services, a lack of culturally appropriate patient education tools, communication barriers, and deep-seated mistrust of the health care system.^5^ Socioeconomic challenges, such as lower socioeconomic status and lower education level, along with limited health care provider experience with Indigenous populations, further worsen these inequities.^5^ Geographic isolation also contributes to reduced access to healthcare resources.^68^ Moreover, anti-Indigenous racism, often reinforced by persistent harmful stereotypes, remains a critical and ongoing driver of health disparities in Canada.^7^

As a result of the aforementioned health inequities, a major challenge for Indigenous peoples in Canada is reduced access to primary care compared with non-Indigenous Canadians,^69^ which reduces opportunities for early detection and management of CKD and can result in more rapid progression to kidney failure. This may in turn account for the increased kidney failure incidence rates among Indigenous patients in Canada.

Furthermore, non-Indigenous research often focuses on didactic techniques and materials for patient education, rather than co-created tools.^70^ This approach is not consistent with Indigenous ways of relational learning and collaborative knowledge sharing.^71^ Also, because Indigenous literacy and numeracy scores are often lower than that of the general population, existing educational materials may be provided in an inaccessible manner.^72^ Consequently, Indigenous patients with kidney disease may be less likely to engage in their own care, which may contribute to worse outcomes.^73^ Collaborative development of educational tools and interventions should go beyond consultation by empowering Indigenous communities to engage in health care decisions and incorporating Indigenous spirituality and traditions within medical practice.^74^ Nevertheless, co-developed approaches are exceedingly rare in kidney care and need to be encouraged.^70^

Additionally, studies evaluating quality of life among Indigenous dialysis patients have demonstrated that relocation to urban centers for treatment has negative implications, including cultural isolation, alienation from family and friends, somatic issues, psychosocial issues, loss of independence and role adjustment.^75^^,^^76^ Moreover, Indigenous dialysis patients often report feeling disconnected from their community even if still living with them because dialysis affects their ability to attend important cultural events and prevents patients from fulfilling their previous role in the community.^77^, ^78^, ^79^

Proposed strategies to improve care for Indigenous patients include the development of Indigenous-led care models, increasing the recruitment and training of Indigenous health care professionals, and providing cultural safety training for non-Indigenous staff.^67^ Efforts should also focus on expanding access to home dialysis, guided by a “Home Dialysis First” approach, alongside increased use of telemedicine and greater financial support for primary care and home dialysis services within communities.^8^ Finally, expanding hemodialysis services in rural communities would help minimize the need for patients who are not eligible for home dialysis to relocate for treatment.

This is the first scoping review to examine health inequities among Indigenous peoples treated with dialysis in Canada, bringing novelty to the literature. We have examined disparities in multiple key clinical outcomes between Indigenous and non-Indigenous dialysis patients, further strengthening our findings. Although this scoping review offers valuable insights, a few limitations in the existing literature on dialysis among Canadian Indigenous peoples should be noted. To start, most of the current research has been conducted by non-Indigenous individuals, and very few by Indigenous peoples. Qualitative research involving Indigenous patients could offer deeper insights into the barriers of dialysis in Indigenous communities. Moreover, our search strategy included interventional and quality improvement studies on the topic, but none were found during the screening process, highlighting an important lack in the current literature.

Several limitations to this scoping review should also be noted. The absence of interventional or quality improvement studies prevents us from demonstrating the effectiveness of interventions targeted at improving outcomes for Indigenous dialysis patients. Additionally, there was some degree of variability in results among studies, especially for mortality. This variability may be attributed to differences in the patient populations examined, which includes the pooling of different Indigenous communities. Furthermore, the included studies were from different Canadian provinces with variable sample sizes, which might affect its overall uniformity. Additionally, as with any review, even if a strong search strategy was built with the help of a librarian, there is a possibility that papers might have been missed. As is common in scoping reviews, we did not conduct a formal quality appraisal, which may affect the reliability of some findings. Future research could build on this work by conducting a systematic review with rigorous quality assessment to evaluate the strength of the evidence. Furthermore, this scoping review focused solely on Canadian studies, which limits its international generalizability. We chose to focus our review on Canada as a unique opportunity to assess and inform policy improvements within a system characterized by universal access and government-funded services. The exclusion of kidney transplant studies, already examined in another scoping review, may equally limit the generalizability of our findings.^14^

## Conclusion

Kidney failure requiring dialysis disproportionately affects Indigenous populations across Canada, and Indigenous patients face worse clinical outcomes compared with non-Indigenous patients. A comprehensive and inclusive approach is needed to better understand the Indigenous experience and help identify approaches to mitigate the burden of kidney failure among these patients. Our findings underscore the critical need for Indigenous-led initiatives and culturally appropriate health care models to address disparities affecting Indigenous patients treated with dialysis. We hope these findings will serve as a beacon to engage Indigenous communities in kidney research to optimize clinical outcomes, guide resource utilization, and ensure equitable care for Indigenous patients. It is crucial to engage Indigenous patients, caregivers and community members to lead the development of interventions that will affect them directly.

## Supplementary Materials

Supplementary File (PDF) Items S1, S2; Tables S1, S2.
