# Supplementary material for: Examining Inequities in Clinical Outcomes for Indigenous Patients Treated With Dialysis in Canada: A Scoping Review
Source: Kidney Med. 2025 Sep 16;7(11):101106. doi: 10.1016/j.xkme.2025.101106 (PMC12597283; doi:10.1016/j.xkme.2025.101106)
Supplement: Supplementary File (PDF) — Items S1, S2; Tables S1, S2. [file mmc1.pdf]

Item S1. Search documentation

Total with duplicates	1804
Duplicates removed	
Total without duplicates	

Database	MEDLINE(R) ALL (Ovid)
Database time coverage	1946-present
Date searched	15 July 2024
Total	146

Database	Embase Classic+Embase (Ovid)
Database time coverage	1947-present
Date searched	15 July 2024
Total	231

Database	CINAHL Plus with Fulltext (Ebsco)
Database time coverage	1936-present
Date searched	15 July 2024
Total	81

Database	Scopus
Database time coverage	1970-present
Date searched	15 July 2024
Total	164

Database	Web of Science Core Collection
Database time coverage	1900-present
Date searched	15 July 2024
Total	1082

Database	Bibliography of Indigenous Peoples of North America (Ebsco)
Database time coverage	16th century-present
Date searched	15 July 2024
Total	100

Item S2. Search summary

Filters used for Indigenous people in Canada for Medline, Embase, CINAHL, Scopus. Modified Scopus filter for use in Web of Science.

Citations

Campbell SM, Dorgan M, and Tjosvold L. Filter to Retrieve Studies Related to Indigenous People of Canada in the OVID Medline Database. Geoffrey & Robyn Sperber Health Sciences Library, University of Alberta. Rev. May 5, 2022. https://docs.google.com/document/d/1CpsxwcUFuMmuWNOmpNVOQuHgYruuoKo8E-M_eMNy4XM/edit#heading=h.qi55eeyvgzy9

Campbell, SM, Dorgan, M and Tjosvold, L. Filter to Retrieve Studies Related to Indigenous People of Canada in the OVID EMBASE Database. Geoffrey & Robyn Sperber Health Sciences Library, University of Alberta. Rev. Oct 4, 2021. https://docs.google.com/document/d/1CpsxwcUFuMmuWNOmpNVOQuHgYruuoKo8E-M_eMNy4XM/edit#

Campbell, SM, Dorgan, M and Tjosvold, L. Filter to Retrieve Studies Related to Indigenous People of Canada in the EBSCO CINAHL Database. Geoffrey & Robyn Sperber Health Sciences Library, University of Alberta. Rev. January 20, 2022. https://docs.google.com/document/d/1CpsxwcUFuMmuWNOmpNVOQuHgYruuoKo8E-M_eMNy4XM/edit#heading=h.3jtqozffmty8

Campbell, SM. Filter to Retrieve Studies Related to Indigenous People of Canada the OVID Medline Database. Geoffrey & Robyn Sperber Health Sciences Library, University of Alberta. Rev. August 24, 2022. https://docs.google.com/document/d/1CpsxwcUFuMmuWNOmpNVOQuHgYruuoKo8E-M_eMNy4XM/edit#heading=h.7l876uie1eqa

Ovid MEDLINE(R) ALL <1946 to July 12, 2024>

1 ((exp Indians, North American/ and Canad*.mp.) or Indigenous Canadians/ or exp Inuits/ or exp Health Services, Indigenous/ or exp Ethnopharmacology/ or (Athapaskan or Saulteaux or Wakashan or Cree or Dene or Inuit or Inuk or Inuvialuit* or Haida or Ktunaxa or Tsimshian or Gitxsan or Gitksan or "Nisga'a" or Haisla or Heiltsuk or Oweenkeno or "Kwakwaka'wakw" or "Nuu chah nulth" or "Tsilhqot'in" or Dakelh or "Wet'suwet'en" or Sekani or Dunne-za or Dene or Tahltan or Kaska or Tagish or Tutchone or Nuxalk or Salish or St'at'imc or Stl'atl'imx or Stl'atl'imc or Nlaka'pamux or Okanagan or "Sec wepmc" or Secwepemc or Tlingit or Anishinaabe or Blackfoot or Nakoda or Tasttine or "Tsuu T'ina" or "Tsuut'ina" or "Gwich'in" or Han or Algonquin or Nipissing or Ojibwa or Potawatomi or Innu or Maliseet or "Mi'kmaq" or Micmac or Passamaquoddy or Haudenosaunee or Cayuga or Mohawk or Oneida or Onondaga or Seneca or Tuscarora or Wyandot or Aboriginal* or Indigenous* or Metis or red road or "on reserve" or off-reserve or First Nation or First Nations or Amerindian).mp. or (urban adj3 (Indian* or Native* or Aboriginal*)).mp. or ethnomedicine.mp. or country food*.mp. or residential school*.mp. or ((exp Medicine, Traditional/ or traditional medicine*.mp.) not Chinese.mp.) or exp Shamanism/ or shaman*.mp. or traditional heal*.mp. or traditional food*.mp. or medicine man.mp. or medicine woman.mp. or autochtone*.mp. or (Native* adj1 (man or men or women or woman or boy* or girl* or adolescent* or youth or youths or person* or adult or people* or Indian* or Nation or tribe* or tribal or band or bands)).mp.) and (exp Canada/ or (Canad* or British Columbia or Colombie Britannique or Alberta or Saskatchewan or Manitoba or Ontario or Quebec or Nova Scotia or New Brunswick or Newfoundland or Labrador or Prince Edward Island or Yukon Territory or NWT or Northwest Territories or Nunavut or Nunavik or Nunatsiavut or NunatuKavut).mp.) 9173

2 exp Kidney Failure, Chronic/ 102847

3 renal dialysis/ or hemodiafiltration/ or hemodialysis, home/ or peritoneal dialysis/ or peritoneal dialysis, continuous ambulatory/ 129378

4 exp Kidney Diseases/mo 24816

5 (dialys?s* or h?emodialys?s* or h?emofiltrat* or h?emodiafiltrat* or h?emo dialys?s* or h?emo filtrat* or h?emo diafiltrat* or ((renal* or kidney* or nephro*) adj4 (therap* or support* or chronic or end stage or endstage))).ti,ab,kf. 334329

6 2 or 3 or 4 or 5 382829

7 1 and 6146

Embase Classic+Embase <1947 to 2024 July 12>

1 ((Indigenous People/ or American Indian/ or First Nation/ or Metis/ or Eskimo/ or Inuit/ or Indigenous Health Services/ or exp Ethnopharmacology/ or (Algonquin or Anishinaabe or Assiniboine or Athapaskan or Blackfoot or Cayuga or Chippewa or Cree or Dakelh or Dene or Dunne-za or Gitsxan or Gwich'in or Haida or (Han not (Chinese or China or Tibet*)) or Haisla or Heiltsuk or Haudenosaunee or Kwakwaka'wakw or Inuit or Inuk or ((Iroquios or Iroquoian) not (homeobox or corn)) or Kaska or Ktunaxa or Nlaka'pamux or Nakoda or Nakota or Nuxalk or Maliseet or Meits or Mi'kmaq or Micmac or Mohawk or Nipissing or Nisga'a or Nuu chah nulth or jibwa or Ojibwe or Ojibway or Oji-Cree or Okanagan or Oneida or Onodaga or Oweenkeno or Passamaquoddy or Potawatomi or Salish or Saulteaux or Sec wepmc or Sekani or Seneca or Stl'atlimc or Tagish or Tasttine or Tahltan or Tlingit or Tsimshian or Tsilhqot'in or Tsuu T'inia or Tuscarora or Tutchone or Wakashan or Wyandot).mp. or Aboriginal*.mp. or Indigenous*.mp. or red road.mp. or "on reserve".mp. or off-reserve.mp. or First Nation.mp. or First Nations.mp. or Amerindian.mp. or (urban adj3 (Indian* or Native* or Aboriginal*)).mp. or ethnomedicine.mp. or country food*.mp. or residential school*.mp. or ((exp Medicine, Traditional/ or traditional medicine*.mp.) not Chinese.mp.) or exp Shamanism/ or shaman*.mp. or traditional heal*.mp. or traditional food*.mp. or medicine man.mp. or medicine woman.mp. or autochtone*.mp. or (Native* adj1 (american or man or men or women or woman or boy* or girl* or adolescent* or youth or youths or person* or adult or people* or Indian* or Nation or tribe* or tribal or band or bands)).mp.) and (exp Canada/ or (Canad* or British Columbia or Colombie Britannique or Alberta or Saskatchewan or Manitoba or Ontario or Quebec or Nova Scotia or New Brunswick or Newfoundland or Labrador or Prince Edward Island or Yukon Territory or NWT or Northwest Territories or Nunavut).mp.)) or (Canadian Aboriginal/ not oriental medicine/) or (Innu or Inuvialuit* or Nunavik or Nunatsiavut or NunatKavut or Wet'suwet'en).mp. 11475

2 exp renal replacement therapy/ 259753

3 exp chronic kidney failure/ 174980

4 exp kidney disease/co, dm, et, th [Complication, Disease Management, Etiology, Therapy] 263602

5 (dialys?s* or h?emodialys?s* or h?emofiltrat* or h?emodiafiltrat* or h?emo dialys?s* or h?emo filtrat* or h?emo diafiltrat* or ((renal* or kidney* or nephro*) adj4 (therap* or support* or chronic or end stage or endstage))).ti,ab,kf. 522010

6 2 or 3 or 4 or 5 768124

7 1 and 6231

CINAHL

#	Query	Limiters/Expanders	Last Run Via	Results
S1	(MH "Aboriginal Canadians+") OR (MH "First Nations of Canada") OR (((MH "Eskimos") OR (MH "Native Americans") OR (MH "Indigenous Peoples+") or (MH "Health Services, Indigenous") or (MH "Indigenous Health") or (MH “Ethnopharmacology”) or Athapaskan or Saulteaux or Wakashan or Cree or Dene or Inuit or Innu or Inuk or Inuvialuit* or Haida or Ktunaxa or Tsimshian or Gitsxan or “Nisga'a” or Haisla or Heiltsuk or Oweenkeno or “Kwakwaka'wakw” or “Nuu chah nulth” or “Tsilhqot'in” or Dakelh or “Wet'suwet'en” or Sekani or “Dunne-za” or Dene or Tahltan or Kaska or Tagish or Tutchone or Nuxalk or Salish or “Stl'atlimc” or “Nlaka'pamux” or Okanagan or “Sec wepmc” or Tlingit or Anishinaabe or Blackfoot or Nakoda or Tasttine or	Expanders - Apply equivalent subjects<br>Search modes - Proximity	Interface - EBSCOhost Research Databases<br>Search Screen - Advanced Search<br>Database - CINAHL Plus with Full Text	5,374

	“Tsuu T'inia” or “Gwich'in” or Han or Tagish or Tutchone or Algonquin or Nipissing or Ojibwa or Potawatomi or Innu or Maliseet or “Mi'kmaq” or Micmac or “Mic mac” or Passamaquoddy or Haudenosaunee or Cayuga or Mohawk or Oneida or Onodaga or Seneca or Tuscarora or Wyandot or Aboriginal* or Indigenous* or Metis or “red road” or "on reserve" or “off reserve” or “First Nation” or “First Nations” or Amerindian or (urban N3 (Indian* or Native* or Aboriginal*)) or ethnomedicine or “country food*” or “residential school*” or ((MH “Medicine, Traditional”) or "traditional medicine*") not Chinese) or (MH“Shamanism”) or shaman* or “traditional heal*” or “traditional food*” or “medicine man” or “medicine woman” or autochtone* or (Native* N1 (American* or man or men or women or woman or boy* or girl* or adolescent* or youth or			

	youths or person* or adult or people* or Indian* or Nation or Nations or tribe* or tribal or band or bands or elder or elders or patient*)))) AND (((MH "Canada+") or (Canad* or "British Columbia" or "Colombie Britannique" or Alberta or Saskatchewan or Manitoba or Ontario or Quebec or "Nova Scotia" or "New Brunswick" or Newfoundland or Labrador or "Prince Edward Island" or "Yukon Territory" or NWT or "Northwest Territories" or Nunavut or Nunavik or Nunatsiavut or NunatuKavut)))			
S2	(MH "Dialysis+")	Expanders - Apply equivalent subjects<br>Search modes - Proximity	Interface - EBSCOhost Research Databases<br>Search Screen - Advanced Search<br>Database - CINAHL Plus with Full Text	27,337
S3	(MH "Kidney Failure, Chronic+")	Expanders - Apply equivalent subjects<br>Search modes - Proximity	Interface - EBSCOhost Research Databases<br>Search Screen - Advanced Search<br>Database - CINAHL Plus with Full Text	24,861
S4	(MH "Kidney	Expanders - Apply equivalent subjects	Interface - EBSCOhost Research Databases	5,804

	Diseases+/MO")	Search modes - Proximity	Search Screen - Advanced Search<br>Database - CINAHL Plus with Full Text	
S5	TI ((dialys#s* OR h#emodialys#s* OR h#emofiltrat* OR h#emodiafiltrat* OR "h#emo dialys#s*" OR "h#emo filtrat*" OR "h#emo diafiltrat*" OR ((renal* OR kidney* OR nephro*) N4 (therap* OR support* OR chronic OR "end stage" OR endstage)))) OR AB ((dialys#s* OR h#emodialys#s* OR h#emofiltrat* OR h#emodiafiltrat* OR "h#emo dialys#s*" OR "h#emo filtrat*" OR "h#emo diafiltrat*" OR ((renal* OR kidney* OR nephro*) N4 (therap* OR support* OR chronic OR "end stage" OR endstage))))	Expanders - Apply equivalent subjects<br>Search modes - Proximity	Interface - EBSCOhost Research Databases<br>Search Screen - Advanced Search<br>Database - CINAHL Plus with Full Text	69,049
S6	S2 OR S3 OR S4 OR S5	Expanders - Apply equivalent subjects<br>Search modes - Proximity	Interface - EBSCOhost Research Databases<br>Search Screen - Advanced Search<br>Database - CINAHL Plus with Full Text	82,875
S7	S1 AND S6	Expanders - Apply equivalent subjects	Interface - EBSCOhost Research Databases	81

		Search modes - Proximity	Search Screen - Advanced Search<br>Database - CINAHL Plus with Full Text	

Scopus

(TITLE-ABS-KEY (athapaskan OR saulteaux OR wakashan OR cree OR dene OR inuit OR inuk OR inuvialuit* OR haida OR ktunaxa OR tsimshian OR gitsxan OR {Nisga'a} OR haisla OR heiltsuk OR oweenkeno OR {Kwakwaka'wakw} OR {Nuu chah nulth} OR {Tsilhqot'in} OR dakelh OR {Wet'suwet'en} OR sekani OR {dunne-za} OR dene OR tahltan OR kaska OR tagish OR tutchone OR nuxalk OR salish OR {stl'atlimc} OR {nlaka'pamux} OR okanagan OR {Sec wepmc} OR tlingit OR anishinaabe OR blackfoot OR nakoda OR tasttine OR {Tsuu T'ina} OR "Tsuut'ina" OR {Gwich'in} OR (han AND NOT chinese) OR tagish OR tutchone OR algonquin OR nipissing OR ojibwa OR potawatomi OR innu OR maliseet OR "Mi'kmaq" OR micmac OR passamaquoddy OR haudenosaunee OR cayuga OR mohawk OR oneida OR onodaga OR seneca OR tuscarora OR wyandot OR aboriginal* OR indigenous* OR metis OR {red road} OR {on reserve} OR {off-reserve} OR {First Nation} OR {First Nations} OR "Original People*" OR indigenous* OR amerindian OR (urban W/3 (indian* OR native* OR aboriginal*)) OR {country food*} OR {residential school*} OR (({traditional medicine*} OR {traditional heal*} OR ethnomedicine*) AND NOT (korea* OR chinese)) OR shaman* OR {traditional food*} OR {medicine man} OR {medicine men} OR "medicine woman" OR {medicine women} OR autochtone* OR (native* W/1 (man OR men OR women OR woman OR boy* OR girl* OR adolescent* OR youth OR youths OR person* OR adult OR patient OR patients OR elder OR elders OR elderly OR people* OR indian* OR nation OR tribe* OR tribal OR band OR bands))) AND TITLE-ABS-KEY (canad* OR "British Columbia" OR {Colombie Britannique} OR alberta OR saskatchewan OR manitoba OR ontario OR quebec OR {nova scotia} OR {New Brunswick} OR newfoundland OR labrador OR {Prince Edward Island} OR "Yukon" OR nwt OR {Northwest Territories} OR nunavut OR nunavik OR nunatsiavut OR nunatukavut)) AND ((TITLE-ABS-KEY (dialys?s* OR h?emodialys?s* OR h?emofiltrat* OR h?emodiafiltrat* OR "h?emo dialys?s*" OR "h?emo filtrat*" OR "h?emo diafiltrat*")) OR (TITLE-ABS-KEY ((renal* OR kidney* OR nephro*) W/4 (therap* OR support* OR chronic OR "end stage" OR endstage)))) n=164

Web of Science

Web of Science Search Strategy (v0.1)

Database: Web of Science Core Collection

Entitlements:

- WOS.IC: 1993 to 2024

- WOS.CCR: 1985 to 2024

- WOS.SCI: 1900 to 2024

- WOS.AHCI: 1975 to 2024

- WOS.BHCI: 2005 to 2024

- WOS.BSCI: 2005 to 2024

- WOS.ESCI: 2005 to 2024

- WOS.ISTP: 1990 to 2024

- WOS.SSCI: 1900 to 2024

- WOS.ISSHP: 1990 to 2024

Searches:

1: ((TS=((Athapaskan OR Saulteaux OR Wakashan OR Cree OR Dene OR Inuit OR Inuk OR Inuvialuit* OR Haida OR Ktunaxa OR Tsimshian OR Gitxsan OR Gitksan OR Nisga'a OR Haisla OR Heiltsuk OR Oweenkeno OR Kwakwaka'wakw OR "Nuu chah nulth" OR Tsilhqot'in OR Dakelh OR Wet'suwet'en OR Sekani OR Dunne-za OR Dene OR Tahltan OR Kaska OR Tagish OR Tutchone OR Nuxalk OR Salish OR St'at'imc OR Stl'atl'imx OR Stl'atl'imc OR Nlaka'pamux OR Okanagan OR "Sec wepmc" OR Secwepemc OR Tlingit OR Anishinaabe OR Blackfoot OR Nakoda OR Tasttine OR "Tsuu T'ina" OR Tsuut'ina OR Gwich'in OR Han OR Algonquin OR Nipissing OR Ojibwa OR Potawatomi OR Innu OR Maliseet OR Mi'kmaq OR Micmac OR Passamaquoddy OR Haudenosaunee OR Cayuga OR Mohawk OR Oneida OR Onondaga OR Seneca OR Tuscarora OR Wyandot OR Aboriginal* OR Indigenous* OR Metis OR "red road" OR "on reserve" OR off-reserve OR "First Nation" OR "First Nations" OR Amerindian) OR (urban NEAR/3 (Indian* OR Native* OR Aboriginal*)) OR ethnomedicine OR "country food*" OR "residential school*"))) OR TS=((Shamanism OR shaman* OR "traditional heal*" OR "traditional food*" OR "medicine man" OR "medicine woman" OR autochtone*)) OR TS=((Native* NEAR/1 (man OR men OR women OR woman OR boy* OR girl* OR adolescent* OR youth OR youths OR person* OR adult OR people* OR Indian* OR Nation OR

tribe* OR tribal OR band OR bands))) Date Run: Mon Jul 15 2024 13:42:43 GMT-0400 (Eastern Daylight Time) Results: 264143

2: TS=((dialys$s* OR hemodialys$s* OR haemodyals$s OR h$emofiltrat* OR h$emodiafiltrat* OR "hemo dialys$s*" OR "haemo dyalys$s" OR "h$emo filtrat*" OR "h$emo diafiltrat*" OR ((renal* OR kidney* OR nephro*) NEAR/4 (therap* OR support* OR chronic OR "end stage" OR endstage)))) Date Run: Mon Jul 15 2024 13:45:48 GMT-0400 (Eastern Daylight Time) Results: 400674

3: #2 AND #1 Date Run: Mon Jul 15 2024 13:46:00 GMT-0400 (Eastern Daylight Time) Results: 1082

Bibliography of Indigenous Peoples of North America

#	Query	Limiters/Expanders	Last Run Via	Results
S1	TI ((dialys#s* OR h#emodialys#s* OR h#emofiltrat* OR h#emodiafiltrat* OR "h#emo dialys#s*" OR "h#emo filtrat*" OR "h#emo diafiltrat*" OR ((renal* OR kidney* OR nephro*) N4 (therap* OR support* OR chronic OR "end stage" OR endstage)))) OR AB ((dialys#s* OR h#emodialys#s* OR h#emofiltrat* OR h#emodiafiltrat* OR "h#emo dialys#s*" OR "h#emo filtrat*" OR "h#emo diafiltrat*" OR ((renal* OR kidney* OR	Expanders - Apply related words; Apply equivalent subjects<br>Search modes - Proximity	Interface - EBSCOhost Research Databases<br>Search Screen - Advanced Search<br>Database - Bibliography of Indigenous Peoples in North America	100

	nephro*) N4 (therap* OR support* OR chronic OR "end stage" OR endstage))))			

Table S1. Preferred Reporting Items for Systematic reviews and Meta-Analyses extension for Scoping Reviews (PRISMA-ScR) Checklist

SECTION	ITEM	PRISMA-ScR CHECKLIST ITEM	REPORTED ON PAGE #
**TITLE**			
Title	1	Identify the report as a scoping review.	1
**ABSTRACT**			
Structured summary	2	Provide a structured summary that includes (as applicable): background, objectives, eligibility criteria, sources of evidence, charting methods, results, and conclusions that relate to the review questions and objectives.	2
**INTRODUCTION**			
Rationale	3	Describe the rationale for the review in the context of what is already known. Explain why the review questions/objectives lend themselves to a scoping review approach.	3
Objectives	4	Provide an explicit statement of the questions and objectives being addressed with reference to their key elements (e.g., population or participants, concepts, and context) or other relevant key elements used to conceptualize the review questions and/or objectives.	4
**METHODS**			
Protocol and registration	5	Indicate whether a review protocol exists; state if and where it can be accessed (e.g., a Web address); and if available, provide registration information, including the registration number.	N/A
Eligibility criteria	6	Specify characteristics of the sources of evidence used as eligibility criteria (e.g., years considered, language, and publication status), and provide a rationale.	4 and Table S2

SECTION	ITEM	PRISMA-ScR CHECKLIST ITEM	REPORTED ON PAGE #
Information sources*	7	Describe all information sources in the search (e.g., databases with dates of coverage and contact with authors to identify additional sources), as well as the date the most recent search was executed.	5
Search	8	Present the full electronic search strategy for at least 1 database, including any limits used, such that it could be repeated.	Item S1 and Item S2
Selection of sources of evidence†	9	State the process for selecting sources of evidence (i.e., screening and eligibility) included in the scoping review.	6 and Table S2
Data charting process‡	10	Describe the methods of charting data from the included sources of evidence (e.g., calibrated forms or forms that have been tested by the team before their use, and whether data charting was done independently or in duplicate) and any processes for obtaining and confirming data from investigators.	6
Data items	11	List and define all variables for which data were sought and any assumptions and simplifications made.	6
Critical appraisal of individual sources of evidence§	12	If done, provide a rationale for conducting a critical appraisal of included sources of evidence; describe the methods used and how this information was used in any data synthesis (if appropriate).	N/A
Synthesis of results	13	Describe the methods of handling and summarizing the data that were charted.	7
**RESULTS**			
Selection of sources of	14	Give numbers of sources of evidence screened, assessed for eligibility, and included in the review, with reasons for exclusions at each	Figure 1

SECTION	ITEM	PRISMA-ScR CHECKLIST ITEM	REPORTED ON PAGE #
evidence		stage, ideally using a flow diagram.	
Characteristics of sources of evidence	15	For each source of evidence, present characteristics for which data were charted and provide the citations.	Table 1
Critical appraisal within sources of evidence	16	If done, present data on critical appraisal of included sources of evidence (see item 12).	N/A
Results of individual sources of evidence	17	For each included source of evidence, present the relevant data that were charted that relate to the review questions and objectives.	Table 1
Synthesis of results	18	Summarize and/or present the charting results as they relate to the review questions and objectives.	7-14 and Figure 2
**DISCUSSION**			
Summary of evidence	19	Summarize the main results (including an overview of concepts, themes, and types of evidence available), link to the review questions and objectives, and consider the relevance to key groups.	14-16
Limitations	20	Discuss the limitations of the scoping review process.	17
Conclusions	21	Provide a general interpretation of the results with respect to the review questions and objectives, as well as potential implications and/or next steps.	17-18
**FUNDING**			
Funding	22	Describe sources of funding for the included sources of evidence, as well as sources of funding for the scoping review. Describe the role of the funders of the scoping review.	19

PRISMA-ScR = Preferred Reporting Items for Systematic reviews and Meta-Analyses extension for Scoping Reviews.

† A more inclusive/heterogeneous term used to account for the different types of evidence or data sources (e.g., quantitative and/or qualitative research, expert opinion, and policy documents) that may be eligible in a scoping review as opposed to only studies. This is not to be confused with *information sources* (see first footnote).
‡ The frameworks by Arksey and O'Malley (6) and Levac and colleagues (7) and the JBI guidance (4, 5) refer to the process of data extraction in a scoping review as data charting.
§ The process of systematically examining research evidence to assess its validity, results, and relevance before using it to inform a decision. This term is used for items 12 and 19 instead of "risk of bias" (which is more applicable to systematic reviews of interventions) to include and acknowledge the various sources of evidence that may be used in a scoping review (e.g., quantitative and/or qualitative research, expert opinion, and policy document).

From: Tricco AC, Lillie E, Zarin W, O'Brien KK, Colquhoun H, Levac D, et al. PRISMA Extension for Scoping Reviews (PRISMAScR): Checklist and Explanation. Ann Intern Med. 2018;169:467–473. doi: 10.7326/M18-0850.

Table S2. Inclusion and Exclusion Criteria

Category	Inclusion Criteria	Exclusion Criteria
Study population	Patients with kidney failure (defined as eGFR < 15 mL/min/1.73m^2 or undergoing dialysis and Canadian Indigenous patients (First Nations, Inuit, and Métis)	Studies assessing only patients with kidney transplantation and/or studies not including Canadian Indigenous patients
Type of studies	Empirical qualitative, quantitative and mixed methods studies	Animal studies, theoretical papers, reviews, opinion pieces, patient narratives
Language	No limit	N/A
Countries	Canada	N/A
Years	No limit	N/A
Focus of studies	Data related to incidence, mortality, treatment complications, access to care and/or quality of life	No data related to incidence, mortality, treatment complications, access to care and/or quality of life
